# Severe Anasarca as a Cause for Stroke-Like Symptoms

**DOI:** 10.51894/001c.166005

**Published:** 2026-07-31

**Authors:** Akshay Sadeeshkumar, Yash Ashara, Marc Feldman

**Affiliations:** 1 College of Osteopathic Medicine Michigan State University

## 118

### Introduction

Stroke mimics pose a significant diagnostic challenge, particularly among patients with multiple vascular comorbidities that independently increase baseline stroke risk. While metabolic and electrolyte imbalances such as hyperkalemia are well-documented causes of transient neuromuscular weakness in dialysis patients, the role of diffuse edema and anasarca as contributors to a stroke-like presentation remains underexplored.

### Case Description

The patient is a 32-year-old female with a history of diabetes mellitus type 1, hypertension, chronic pancreatitis, chronic kidney disease stage 4, and prior infarct of the right cerebral peduncle, who presented to the ER with significant generalized edema and acute weakness of her right upper and lower extremities. Initial suspicions for stroke led us to neuroimaging; however, head CT w/o contrast and subsequent MRI of the brain and spine revealed no acute infarction, demyelination, or compressive pathology. Laboratory evaluation upon admission showed elevated blood urea nitrogen (27) and creatinine (3.19), with her BUN and creatinine spiking on 3rd day of admission at 35 and 3.94 respectively; sodium and potassium were consistently within normal limits. Physical examination demonstrated severe generalized edema across her body and head, with MRI signifying diffuse paraspinal edema. With initiation of aggressive diuresis and eventual hemodialysis on the 5th day of admission, the patient’s anasarca, alongside her unilateral weakness, improved markedly within days.

### Discussion

Unilateral neuromuscular weakness remains an uncommon, yet debilitating presentation of anasarca. In this case, severe diffuse paraspinal edema may have contributed to transient focal neuropathic dysfunction; with treatment focused on reducing her fluid overload, we observed a noticeable improvement in the patient’s neuromuscular strength. Alternative etiologies such as post-stroke syndrome, diabetic neuropathy, and uremic neuropathy were considered. However, the lateralization inconsistent with her prior infarct, the intense and acute appearance of her weakness, and the rapidly resolving nature of it with aggressive diuresis and dialysis made them less likely. There should be a consideration of transient neuropathic weakness secondary to paraspinal edema in CKD patients presenting with unilateral weakness with anasarca after ruling out obvious neurological deficits.

